# Fast response to human voices in autism

**DOI:** 10.1038/srep26336

**Published:** 2016-05-19

**Authors:** I-Fan Lin, Trevor R. Agus, Clara Suied, Daniel Pressnitzer, Takashi Yamada, Yoko Komine, Nobumasa Kato, Makio Kashino

**Affiliations:** 1 NTT Communication Science Laboratories, 3-1 Morinosato Wakamiya, Atsugi, Kanagawa, 243-0198 Japan; 2 Department of Human Sciences, Tokyo Metropolitan University, 1-1 Minami-Osawa, Hachioji, Tokyo, 192-0397 Japan; 3 School of Creative Arts, Queen’s University Belfast, Belfast, BT7 1NN, United Kingdom; 4 Département Action et Cognition en Situation Opérationnelle, Institut de Recherche Biomédicale des Armées, 91223 Brétigny sur Orge, France; 5 CNRS UMR 8248, Ecole normale supérieure, 29 rue d’Ulm, 75005 Paris, France; 6 Medical Institute of Developmental Disabilities Research, Showa University, Kitakarasuyama 6-11-11, Setagaya, Tokyo, 157-8577 Japan; 7 ATR Brain Information Communication Research Laboratory Group, Hikaridai 2-2-2, Sourakugun Seikacho, Kyoto, 619-0237 Japan; 8 Department of Information Processing, Interdisciplinary Graduate School of Science and Engineering, Tokyo Institute of Technology, 4259 Nagatsuta, Midori, Yokohama, Kanagawa, 226-8503 Japan

## Abstract

Individuals with autism spectrum disorders (ASD) are reported to allocate less spontaneous attention to voices. Here, we investigated how vocal sounds are processed in ASD adults, when those sounds are attended. Participants were asked to react as fast as possible to target stimuli (either voices or strings) while ignoring distracting stimuli. Response times (RTs) were measured. Results showed that, similar to neurotypical (NT) adults, ASD adults were faster to recognize voices compared to strings. Surprisingly, ASD adults had even shorter RTs for voices than the NT adults, suggesting a faster voice recognition process. To investigate the acoustic underpinnings of this effect, we created auditory chimeras that retained only the temporal or the spectral features of voices. For the NT group, no RT advantage was found for the chimeras compared to strings: both sets of features had to be present to observe an RT advantage. However, for the ASD group, shorter RTs were observed for both chimeras. These observations indicate that the previously observed attentional deficit to voices in ASD individuals could be due to a failure to combine acoustic features, even though such features may be well represented at a sensory level.

The hallmarks of autism spectrum disorders (ASD) are repetitive behaviors combined with impairments in communication and social engagement. While neurotypical (NT) infants tend to orient toward their mothers’ voices^1^, infant-directed speech^2^, and their own names^3^, ASD children do not show the same preference toward their mothers’ voices^4^, and they respond less to their names than other NT children^5^,^6^,^7^. Clinical observations also suggest that ASD children are indifferent toward the sounds of speech^8^. If ASD children do not orient to human voices, they may fail to learn the sophisticated social signals that their NT counterparts rely on to communicate. In addition, based on the hypothesis that the earliest phases of language acquisition requires social interaction, the absence of preference to human voices may jeopardize the development of language in ASD children^9^,^10^,^11^. Later in life, ASD adults still exhibit abnormal voice processing: they have difficulties extracting mental state information from voices^12^, and their performance in this task is positively correlated with verbal intelligence quotient (IQ)^13^.

Recent studies have suggested two possible explanations for the observed indifference to voices in ASD individuals. Firstly, it might result from a sensory deficit specific for voices. Compared to NT adults, ASD adults show less activation in the superior temporal sulcus (STS), the secondary auditory area that is the locus of the main voice-selective region in human brain^14^. Alternately, the deficit might be associated with the reward and attention system for voices. One imaging study did reveal an under-connectivity between the posterior STS and the dopaminergic reward pathway and amygdala in ASD children^15^. Favoring the under-rewarded voices hypothesis, two event-related brain potentials (ERPs) studies found normal auditory sensory ERPs for both speech and non-speech stimuli, such as pure tones and complex tones, but reduced involuntary attention to speech (as indexed in P3a in the mismatch negativity component) in ASD individuals^16^,^17^.

The present study was designed to investigate, behaviorally, how high-functioning ASD adults react to human voices when they are *instructed to pay attention* to the auditory stimuli. We used reaction times (RTs) to evaluate the time course of processing of natural sounds, including voices. The sound set was designed to remove all acoustic cues apart from timbre, while retaining ecological validity. We chose to contrast carefully-matched sung vowels and musical instrument sounds. Moreover, to explore the acoustic substrate of voice recognition in both the ASD and NT groups, we created auditory chimeras retaining only the temporal or spectral components of the voices (see Methods below and reference^18^).

To investigate the detection and recognition processes of voices and non-voice sounds in ASD individuals, we designed an experiment comprising a simple RT task and four go/no-go tasks. In the simple RT task, participants listened to successive, brief auditory stimuli (voices, sounds of strings, and auditory chimeras) and were asked to respond as fast as possible to all stimuli. In the go/no-go tasks, they were asked to react as fast as possible only to a designated class of target stimuli (for different blocks: voices, sounds of strings, or auditory chimeras) while ignoring other distracting stimuli (sounds of bassoon, clarinet, oboe, piano, saxophone, trombone, and trumpet). Strings were chosen as a target class in an attempt to have a family of sounds with some diversity (the strings class included bowed cello and violin sounds) and a formant structure somewhat similar to voices (the voice class included the vowels /a/ and /i/). This choice follows on a previous study^18^ that used the same sound set and procedure, but only tested NT individuals. There, it was shown that voices were processed faster in the go/no-go task compared to strings. Importantly, no voice advantage was found for the simple RT task, suggesting that the sound set was well-matched acoustically. The go/no-go RT difference was ascribed to recognition processes. Finally, chimeras were chosen to impose an even closer control of the acoustic characteristics of the target classes. Chimeras retained exactly either the temporal or the spectral components of voices. In the previous study for NT adults^18^, such chimeras were found to be processed slower than voices. In summary, for NT individuals, previous results show that voices are processed faster than acoustically-comparable sound classes, and that only the full combination of temporal and spectral components of the vocal sounds triggers faster processing.

Predictions can be made about the ASD group tested here. The first obvious possibility is that they show no evidence of a fast, low-level recognition process for voices, consistent with their high-level deficit with speech stimuli. This is not what we observed: the fast recognition process was present, and it was even faster for ASD adults compared to NT adults. Another possibility is that the low-level representation of vocal sounds is comparable for ASD individuals compared to NT individuals, but that some higher processes sitting between voice representation and speech perception are impaired for the ASD group. The auditory chimeras allowed us to test specifically for one such higher-level impairment, which had previously been termed “weak central coherence”^19^. In many perceptual tasks, ASD individuals were found to have superior local processing but impaired global processing compared to NT individuals^19^. Here, if ASD individuals reacted to the vocal features contained within the chimeras without holistically combining vocal and non-vocal features, then they may in fact show a better (faster) RT for chimeras compared to NT individuals. This is what we observed. In the remaining of the paper, we present in details the experimental results and discuss how our findings relate to impairments in speech processing for ASD adults.

## Methods

### Participants

We recruited 12 ASD adults and 12 NT adults. Informed consent was obtained from all individual participants included in the study. We verified that all but one of the participants had normal hearing, defined as pure-tone hearing thresholds of 20 dB HL or less at audiometric frequencies between 500 and 8000 Hz. The data of one NT participant was excluded due to his hearing loss. The remaining 12 ASD and 11 NT participants were IQ-matched (using WIAS-III or WAIS-R)^20^,^21^,^22^ and age-matched. Table 1 provided detailed information about the participants. All procedures were conducted in accordance with the Declaration of Helsinki and approved by the Ethics Committee of the NTT Communication Science Laboratories. Participants were naive to the purposes of the study, and they were paid for their time.

The diagnosis of ASD was based on a consensus reached by three experienced psychiatrists and one psychologist according to the criteria of Diagnostic and Statistical Manual of Mental Disorders (DSM-IV-TR)^23^. Two detailed interviews were conducted independently by a psychiatrist and a clinical psychologist, belonging to the team at Karasuyama Hospital. Criteria included the participant’s developmental history, present illness, past history, and family history. Of the 12 ASD participants, 6 were diagnosed with Asperger’s syndrome, 4 were diagnosed as having high-functioning autism, and 2 were diagnosed as exhibiting Pervasive Developmental Disorder-Not Otherwise Specified (PDD-NOS). None of the participants met the DSM-IV-TR criteria for any other psychiatric disorders.

### Auditory stimuli

The target stimuli included voices (a male voice singing the vowels /a/ or /i/) and strings (sounds of violin and cello). The distracting stimuli included other musical instruments (sounds of bassoon, clarinet, oboe, piano, saxophone, trombone, and trumpet). All of them were single musical notes extracted from the RWC Music Database^24^ covering a common pitch range of 12 semitones between A3 and G#4. Each note was edited to a separate sound file, truncated to a duration of 250 ms (with a cosine fade-in ramp of 5 ms and a cosine fade-out ramp of 50 ms) then normalized to have the same root-mean-square (rms) power.

The auditory chimeras were composed of either the temporal features of voices with the spectral features of sounds of strings (henceforth termed temporal chimeras), or the spectral features of voices with the temporal features of sounds of strings (termed spectral chimeras). Sound examples of chimeras can be found at http://audition.ens.fr/chimeras/. Briefly, we used an auditory model to process the sounds in 64 overlapping frequency bands. The average amplitude in each band defined the auditory spectral profile (i.e., excitation patterns^25^) of each sound. The time waveform in each band contained all other cues, which were temporal and not spectral. We then created chimeras by imposing the spectral profile of one sound onto a different sound. For example, we imposed the spectral profile of voices onto the temporal profile of strings to obtain the spectral chimeras. All further details to generate the auditory chimeras are specified in reference^18^.

Auditory stimuli were played through an M-AUDIO FireWire 410 audio interface at a 44.1 kHz sample-rate. They were presented to both ears simultaneously, through Sennheiser HDA 200 Headphones. The average sound pressure level of all the stimuli and a calibrating tone (1000-Hz pure tone) was normalized to 58.8 dB(A) as measured by a Brüel & Kjær sound level meter (Wide Range Measuring Amplifiers, Types 2610). Listeners were tested individually in a sound-insulated booth, and their responses were recorded through a custom-made response box.

### Procedure

There were five blocks in the experiment. The first block was always a simple RT task: participants listened to successive brief sounds, which consisted of voices, sounds of strings, and two kinds of auditory chimeras. Participants were instructed to respond as fast as possible to each sound. After each response, a random inter-trial silent gap was introduced, with a duration between 1000 ms and 1800 ms, in order to prevent anticipation of the next stimulus. The subsequent four blocks were go/no-go tasks: participants again listened to successive brief sounds, but this time they consisted of a designated category of target stimuli (voices, sounds of strings, or either kind of auditory chimeras, on different blocks) randomly interspersed within distracting stimuli (sounds of bassoon, clarinet, oboe, piano, saxophone, trombone, and trumpet). A random inter-trial silent gap was introduced, as in the simple RT task. Participants were instructed to respond as fast as possible to the designated target stimuli only, and to withhold their response for the distracting stimuli. Target stimuli were presented in 53% of the trials. At the beginning of the experiment, participants completed a short (around 5 minutes) simple RT task and a go/no-go task (in which the target type was randomly chosen for each participant) to familiarize them with the procedures. The data of those training blocks were not analyzed.

Before each block, participants were presented with the opportunity to listen freely to the designated target sounds for that block, as well as to the distracting sounds for the go/no-go blocks. When they felt ready to start the block, they pressed the ‘start’ button displayed on the computer screen. After a pause of random duration (1000–1800 ms), the first sound for the block was presented.

In the simple RT task, there were 24 trials (12 notes*2 target subcategory*1 presentations) for each of the four subsequent target categories, resulting in 96 trials. For the go/no-go blocks, the designated target sounds were either voices, or sounds of strings, or either kind of auditory chimeras. Each target category comprised two different subcategories: vowels /a/ and /i/ for the voices category, violin and cello for the strings category, and an arbitrary combination of vowel/string instrument for the chimeras category. The order of these four blocks were randomized for each listener. Each block was formed from 96 target trials (12 notes*2 target subcategory*4 presentations) and 84 distractor trials (12 notes*7 distractor instruments*1 presentation), resulting in 180 trials.

### Data analysis

RTs that were shorter than 100 ms were defined as anticipations and excluded from the analysis. On average, 1.27% and 0.49% of the RTs in the target trials were excluded in this way for the simple RT task and go/no-go task in the ASD and NT groups, respectively. Our custom-made response device had a pre-set upper cut-off limit (1250 ms), so the exclusion rate for longer reaction times had to be estimated by simulated data based on measured data^26^. Based on those simulations, we estimate the exclusion rate for late responses to be on average 0.14% and 0.75% of the RTs in the target trials, for the simple RT task and go/no-go tasks, respectively. Detailing these figures between groups, the simulations showed that in the simple RT tasks, <0.01% and 0.28% of the RTs were excluded in the ASD and NT groups, respectively. In the go/no-go task, 0.26% and 1.23% of the RTs in the target trials were excluded in the ASD and NT groups, respectively.

Because RTs were not normally distributed, all RTs were transformed logarithmically before calculating statistics (e.g., ANOVAs and *t* tests). This includes the means and 95% confidence intervals displayed in the figures, which were converted back to linear time for ease of reading.

## Results

**Figure 1:**
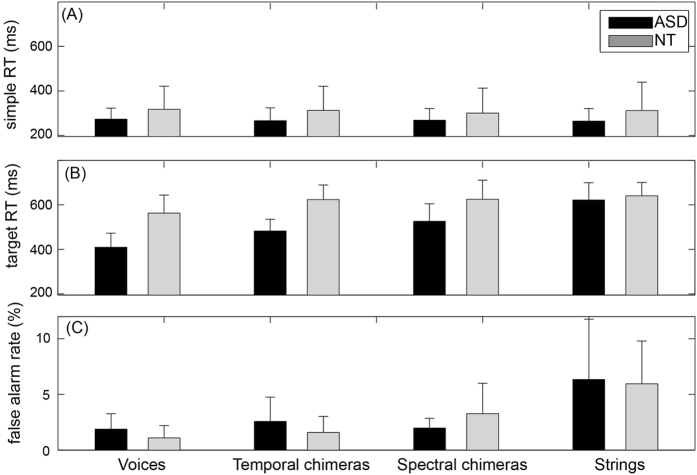
Behavioral data for the different target types in the simple RT task and the go/no-go tasks. Bars indicate the mean over participants and error bars are the ±95% confidence interval. RTs were computed on log-transformed values and displayed back on a linear scale for clarity. (**A**) There was no between-group or between-target difference in RTs in the simple RT task. (**B**) Similar to NT adults (gray bars), ASD adults (black bars) had significantly shorter RTs for voices targets than for strings targets. ASD adults had even shorter RTs for voices than the NT adults. For auditory chimeras (see text), the RTs were as slow as the strings for the NT group, but faster than the strings for the ASD group. Overall, the ASD group had shorter RTs than the control group only in the go/no-go tasks, and only in sounds with vocal acoustic cues (**C**) There was no between-group difference in false alarm rate for the go/no-go tasks.

Figure 1A displays the results in the simple RT task. The average RTs for the NT and ASD groups were 310 ms and 268 ms, respectively. There was no obvious between-group or between-target difference. This observation was confirmed by a two-way mixed ANOVA, with Group (ASD, NT) as a between-subjects factor and Type (voices, strings, temporal chimeras, spectral chimeras) as a within-subjects factor (see Table 2), which did not indicate any significant difference (all p > 0.05). So, when listeners were instructed to respond to sound in general, the two groups were equally fast for all tested types of sounds, vocal or not.

Figure 1B displays the average RTs in the go/no-go tasks, for correct responses on the target trials. Here the RTs for voices were the shortest (averaged RTs were 562 ms and 410 ms in the NT and ASD groups, respectively), while RTs for strings were the longest (averaged RTs were 641 ms and 621 ms in the NT and ASD groups, respectively). The RTs for temporal and spectral chimeras were in between (temporal chimera: averaged RTs were 624 ms and 483 ms in the NT and ASD groups, respectively; spectral chimeras: averaged RTs were 625 ms and 526 ms in the NT and ASD groups, respectively). A two-way mixed ANOVAs was conducted for RTs in the go/no-go tasks, with Group as between-subjects factor and Type as within-subjects factor. The Group and Type factors were significant, as was their interaction (Table 2).

To investigate precisely the effect of Type on each group, we conducted two follow-up one-way ANOVAs with Type as a within-subjects factor, for the two groups separately. For both groups, the factor Type was significant (NT: F(3,30) = 4.612, p = 0.009; ASD: F(3,33) = 33.767, p < 0.001). Post-hoc pair-wise comparisons (with Bonferroni correction) showed that for both groups, RTs were significantly shorter for voices than for strings (t(10) = 3.330, p = 0.046 and t(11) = 9.424, p < 0.001, in the NT and ASD groups, respectively). However, while the two auditory chimeras evoked similar RTs as voices and strings in the NT group (compared to voices: t(10) = 2.866 and 1.990, p = 0.101 and 0.447 for temporal and spectral chimeras, respectively; compared to strings: t(10) = 1.256 and 0.789, p = 1.000 and 1.000 for both chimeras), RTs for the two auditory chimeras were significantly longer than RTs for voices (t(11) = 4.645 and 5.267, p = 0.004 and 0.002 for temporal and spectral chimeras, respectively) but significantly shorter than RTs for strings (t(11) = 6.934 and 3.666, p < 0.001 and 0.022 for temporal and spectral chimeras, respectively) in the ASD group.

To investigate between-group difference for each target type, four *t*-tests were run (due to multiple comparisons, the alpha value should be adjusted to 0.0125 based on Bonferroni correction). They showed significant between-group differences for voices (t(21) = 3.560, p = 0.002) and temporal chimeras (t(21) = 3.934, p < 0.001), but no significant between-group differences for spectral chimeras (t(21) = 2.000, p = 0.059) or strings (t(21) = 0.455, p = 0.654).

We ran an alternate analysis of the RT data, combining both tasks and normalizing each participants’ go/no-go RTs by their simple RTs (Supplementary Information and Supp. Fig. 1). Even though such “difference RTs” cannot be equated to recognition times^27^, they may help cancel out some of the inter-subject variability in RTs. The same conclusions were reached: voices were recognized faster than strings for both groups, and the ASD group recognized voices and temporal chimeras even faster than the NT group.

Finally, we analyzed incorrect responses. Figure 1C displays the false alarm rates in the go/no-go tasks, that is, when participants responded to a non-target sound. The false alarm rate was lowest for voice blocks in both groups (the medians of false alarm rate were 1.2% in both the NT and ASD groups). The false alarm rate was highest for strings blocks in both groups (the medians for false alarm rate were 4.8% and 3.0% for the NT and ASD groups, respectively). The false alarm rate for chimeras blocks was in between (the medians of false alarm rate were 1.2% for temporal chimeras in both groups and 2.4% and 1.8% for spectral chimeras in the NT and ASD groups, respectively). The main finding is that those false alarm rates were all very low. The other finding is that the faster RTs for voices were not due to a speed-accuracy trade-off, as responses to voices were both faster and more accurate.

We next analyzed the RTs for false-alarm trials. Because many participants had zero false alarm responses in some conditions, Friedman’s ANOVAs and Mann-Whitney tests were used. The results of Friedman’s ANOVAs showed that Type was a significant factor for the NT group (*X*^2^(3) = 10.34, p = 0.016) but not significant in the ASD group (*X*^2^(3) = 7.625, p = 0.054). The results of Mann-Whitney tests showed that there was no significant between-group difference for any kind of target types (voices: U = 55, z = −0.709, p = 0.525; strings: U = 80, z = 0.872, p = 0.413; temporal chimeras: U = 57, z = −0.575, p = 0.608; spectral chimeras: U = 78, z = 0.76, p = 0.487).

## Discussion

This study investigated how high-functioning adults with ASD processed vocal sounds when they were instructed to pay attention to such sounds. We found that, similar to NT adults, ASD adults had significantly shorter RTs for voices than for strings. Furthermore, even though both experimental groups had similar performance on non-vocal sounds or on the task not involving voice recognition, ASD adults specifically had shorter RTs for voice recognition compared to NT adults. The auditory chimeras add another element to the between-group comparison. While the NT group did not retain the voice-processing advantage when either temporal or spectral voice-specific information was preserved, the ASD group seemingly did, at least partially, as they exhibited faster RTs for the chimeras compared to non-vocal sounds.

An important preliminary remark when discussing our findings is that the between-group difference in RTs for vocal sounds cannot be explained by between-group differences in motor planning, motor execution, understanding of the task, or other non-sensory abilities. The participants in the ASD group and the control group were matched in IQ, and, crucially, they displayed identical performance for all auditory stimuli in the simple RT task, as well as for sounds of strings in the go/no-go task. Thus, the difference can be assumed to be specific to the sensory processing of vocal sounds.

The simple RT task and go/no-go tasks measured different aspects of auditory processing. For the simple RT task, the results reflect the ability to detect rapidly low-level acoustic information^28^. In some studies, the acoustic properties of the sounds have been found to modulate the simple RTs^29^. The present results, showing no significant difference in simple RTs across the four target types, suggest that targets were well matched acoustically (in terms of e.g. onset time) and equally easy to detect. Moreover, the ASD and NT groups displayed an equal ability to perform the simple RT task.

In contrast, the go/no-go task requires a fast recognition of the sound source, as responses must only be made for targets and not for distractors. The short RTs for voices observed in such a task for NT listeners (present study and Agus *et al*.’s study^18^) are consistent with a fast processing pathway dedicated to voices, as suggested by EEG studies^30^,^31^. It is also consistent with the existence of the voice-selective cortical area in the higher auditory pathways, identified with fMRI studies^32^,^33^. For the NT group, only the natural voices were able to recruit such voice-specific processes: the auditory chimeras produced RTs as slow as the string sounds (present study and Agus *et al*.’s study^18^). This suggests that NT listeners required the full combination of acoustic cues, which are probably represented concurrently in primary auditory cortex^34^,^35^, to benefit from voice-specific processing in sound source recognition.

The results differed markedly between the ASD and NT groups for the go/no-go task. Although previous studies have reported reduced preference toward voices or name calling in children with ASD^4^,^5^,^6^,^7^,^8^, here we showed that when ASD adults were instructed to pay attention to auditory stimuli, their RTs for vocal sounds were in fact faster than for NT adults. This suggests even faster voice processing in ASD adults when they pay attention. In addition, the type of sounds that induced fast processing was different between the ASD and NT groups. The ASD group displayed fast processing for voices as well as for auditory chimeras, especially those containing the temporal components of voices.

These observations are surprising in two ways. First, ASD children have been shown to have less neural activation in the voice-selective cortical area when presented with vocal sounds, which seemingly provides a neural basis for their reduced sensory processing for voices^14^. Second, the processing efficiency of temporal modulated auditory stimuli in ASD individuals is found to be reduced^36^.

We can reconcile our findings with the literature by proposing the following, novel account of the vocal deficit displayed by ASD people observed without directed attention: ASD individuals have a preserved low-level representation of vocal sounds, but do not process them in a holistic manner. The supporting evidence from our results is as follows. In our study, participants received explicit instructions to pay attention to the target sounds including voices, and the ASD group performed at least as well as the NT group. This suggests that their low-level representation of the vocal sounds was preserved. Preserved low-level speech representations have also been put forward by at least some ERP studies that contrast speech and non-speech sounds^16^,^17^. In addition, the ASD group reacted faster to auditory chimeras that included both vocal and non-vocal cues, unlike the NT group. This could be because they processed the sounds in an analytical manner, relying independently on the different acoustic cues to a voice. While such analytical listening may have favored them in our RT task with chimeras, as they may have been oblivious to the conflicting cues present in the sounds, it could also be detrimental when listening to natural speech. Consistent with this speculation, it has been shown that when ASD individuals listen to complex sounds, their neural activity in primary auditory cortex is larger than those for NT individuals but their neural activity in non-primary auditory cortex is lower than those for NT individuals^37^. This interpretation is consistent with the enhanced perceptual functioning theory^38^, which describes the enhanced perception of local features in individuals with autism. It is also consistent with the “weak central coherence” theory^19^. What remains to be investigated is whether the lack of spontaneous orientation to voices^16^,^17^ and the abnormal neural connectivity between vocal processing and reward networks^15^ are a consequence of this lack of holistic processing, whether they may be a cause of it, or whether the two observations are co-occurring but independent.

Finally, why were ASD listeners even faster than NT listeners on the natural voice recognition task? A previous study showed that the reduced attentional shifts to speech observed in ASD children in the passive condition, as indicated by the magnitude of the P3a in an oddball paradigm, is restored in the active condition^39^. One explanation for this finding is that ASD children do not have difficulty in shifting their attention to speech sounds, but rather, they may actively inhibit these responses in normal circumstances. In the go/no-go tasks used here, listeners needed to disinhibit their responses for the target stimuli. When the inhibition to voices was reduced by complying with the experimental instructions, the behavioral performance of the ASD group might therefore be enhanced. Another consideration is that the ASD participants in this study were high-functioning adults, and many of them were actively participating in rehabilitation programs. It is possible that they compensated their indifference to voices by paying special attention to voices, and thus their RTs for voices were shorter than the RTs observed in the control group.

In summary, this study provides evidence that reaction toward voices in the go/no-go tasks was faster in ASD individuals than in NT individuals when they were instructed to pay attention to auditory stimuli. This observation shows that the high-level deficit observed for vocal sounds in ASD are unlikely to be due to degraded sensory representation. Rather, they could indicate impairments in the holistic processing of the complex acoustic cues to voices, and/or in the perceived reward value of the voice.

## Additional Information

**How to cite this article**: Lin, I.-F. *et al*. Fast response to human voices in autism. *Sci. Rep.* **6**, 26336; doi: 10.1038/srep26336 (2016).

## Supplementary Material

Supplementary Information

## Figures and Tables

**Table 1:** Mean-group matching data for the ASD and NT participants (shown as mean ± standard deviation).

**Gender (female:male)**	**ASD**	**NT**	***t*** **test**
	**3:9**	**2:9**	**–**
Age	27.5 ± 7.93	27.27 ± 9.24	p = 0.95
Full IQ	105.33 ± 12.57	108.64 ± 15.63	p = 0.59
Performance IQ	100.58 ± 11.33	104.91 ± 14.79	p = 0.44
Verbal IQ	108.33 ± 15.82	110.45 ± 17.79	p = 0.77
AQ^36^	37.75 ± 4.56	18.45 ± 6.58	p < 0.001

**Table 2:** Results of ANOVAs for RTs in the simple RT task and in the go/no-go tasks.

		**df**	**F**	**p**
Simple RT	Group	1,21	0.856	0.365
	Type	3,63	2.014	0.121
	Group* Type	3,63	1.298	0.283
Target RT	Group	1,21	7.811	0.011*
	Type	3,63	31.566	<0.001***
	Group* Type	3,63	9.422	<0.001***
