# Supplementary material for: Fast response to human voices in autism
Source: Sci Rep. 2016 May 19;6:26336. doi: 10.1038/srep26336 (PMC4872056; doi:10.1038/srep26336)
Supplement: Supplementary Information [file srep26336-s1.pdf]

Supplementary Information

Fast response to human voices in autism

I-Fan Lin[1,2], Trevor R. Agus[3], Clara Suied[4], Daniel Pressnitzer[5], Takashi Yamada[6,7], Yoko Komine[6], Nobumasa Kato[6], Makio Kashino[1,8]

1. NTT Communication Science Laboratories, 3-1 Morinosato Wakamiya, Atsugi, Kanagawa, 243-0198 Japan

2. Department of Human Sciences, Tokyo Metropolitan University, 1-1 Minami-Osawa, Hachioji, Tokyo, 192-0397 Japan

3. School of Creative Arts, Queen's University Belfast, Belfast, BT7 1NN, United Kingdom

4. Département Action et Cognition en Situation Opérationnelle, Institut de Recherche Biomédicale des Armées, 91223 Brétigny sur Orge, France

5. CNRS UMR 8248, Ecole normale supérieure, 29 rue d'Ulm, 75005 Paris, France

Supplementary analysis of the main results: difference RTs

Method

We subtracted the simple RTs to the go/no-go RTs for each subject and sound type, after log-transformation of the respective RTs. When doing so, we observed that two participants for the NT group displayed longer simple RTs than go/no-go RTs for some blocks. This violates the basic assumption that difference RTs are related to recognition times for those two participants. The reason for this behavior are unclear, but it is worth noting that the simple RT task was always performed before the go/no-go tasks, so procedural learning may have played a role. For the supplementary analysis only, these

two listeners were considered as outliers and excluded from the NT group.

Results

Results are shown in Supplementary Figure 1. The pattern of results was highly similar to that observed with go/no-go RTs. We performed the same statistical analyses for the difference RTs as for the go/no-go RTs (Fig. 1B). We first conducted a two-way mixed ANOVA on difference RTs with Group as between-subjects factor and Type as within-subjects factor. The results showed significant effect of Group ($F(1,19)=5.042$, $p=0.037$) and Type ($F(3,57)=40.157$, $p<0.001$) and interaction between Group and Type ($F(3,57)=7.307$, $p<0.001$).

Two follow-up one-way ANOVAs with Type as a within-subjects factor were conducted for the two groups separately. For both groups, the factor Type was significant (NT: $F(3,24)=9.788$, $p<0.001$; ASD: $F(3,33)=33.58$, $p<0.001$). For the NT group, post-hoc pair-wise comparisons with Bonferroni correction showed that difference RTs for voices were significantly smaller than those for strings ($t(8)=4.770$, $p=0.008$). There were no differences between auditory chimeras and strings ($t(8)=0.418$ and 1.799, $p=1$ and 0.658 for temporal and spectral chimeras, respectively), nor between spectral chimeras and voices ($t(8)=2.550$, $p=0.205$) or between the two types of chimeras ($t(8)=1.200$, $p=1$). However, difference RTs for temporal chimeras were significantly smaller than those for voices ($t(8)=4.616$, $p=0.010$). For the ASD group, the same post-hoc pair-wise comparisons showed that difference RTs were significantly smaller for voices than those for strings ($t(11)=9.296$, $p<0.001$) and for temporal and spectral chimeras ($t(11)=7.506$ and 3.696, $p=0.007$ and 0.003, respectively). Difference RTs were significantly larger for strings than those for the temporal and spectral chimeras ($t(11)=4.289$ and 4.887, $p=0.008$ and $p=0.003$, respectively). There was no significant difference between the two types of chimeras ($t(11)=2.237$, $p=0.282$).

For between-group comparison of difference RTs, there was no significant difference for strings ($t(19)=0.623$, $p=0.541$) and spectral chimeras ($t(19)=1.521$, $p=0.145$), but there was significant difference for voices ($t(19)=3.696$, $p=0.002$) and temporal chimeras ($t(19)=3.155$, $p=0.005$).

Discussion

The analysis of difference RTs led to almost identical conclusions as that for go/no-go RTs, with only one noteworthy exception: difference RTs for temporal chimeras were significantly smaller than those for voices in the NT group. This observation is surprising, but it does not affect our conclusion that for NT listeners, only the combination of spectral and temporal components of voices evoked a faster recognition processes.

Acknowledgement

We would like to thank one anonymous Reviewer for suggesting this supplementary analysis.

Supplementary Figure 1

Difference between simple RTs and RTs in the go/no-go tasks after logarithmic transformation for the different target types. Bars indicate the mean over participants and error bars are the ± 95% confidence interval.

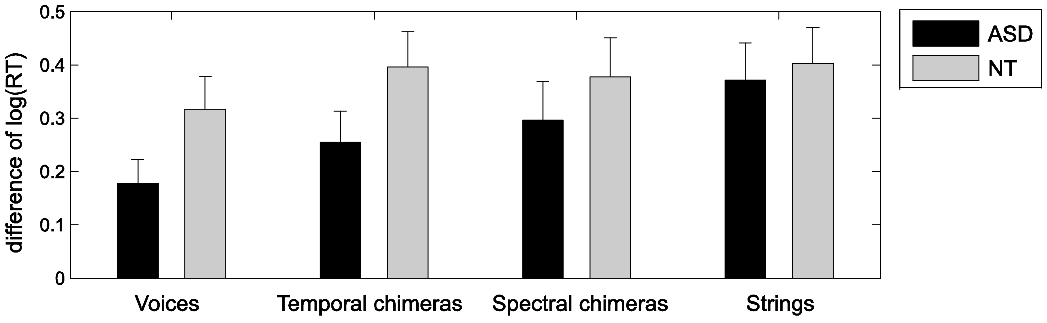

difference of log(RT)
0.5
0.4
0.3
0.2
0.1
0
Voices
Temporal chimeras
Spectral chimeras
Strings
ASD
NT
